# Selecting Hub Genes and Predicting Target Genes of microRNAs in Tuberculosis via the Bioinformatics Analysis

**DOI:** 10.1155/2021/6226291

**Published:** 2021-10-31

**Authors:** Siqi Deng, Shijie Shen, Saeed El-Ashram, Huan Lu, Dan Luo, Guomin Ye, Zhen feng, Bo Zhang, Hui Zhang, Wanjiang Zhang, Jiangdong Wu, Chuangfu Chen

**Affiliations:** ^1^Key Laboratory of Xinjiang Endemic and Ethnic Diseases Cooperated By Education Ministry with Xinjiang Province, Shihezi University, Shihezi 832002, China; ^2^College of Life Science and Engineering, Foshan University, 18 Jiangwan Street, Foshan 528231, Guangdong Province, China; ^3^Faculty of Science, Kafrelsheikh University, Kafr El-Sheikh 33516, Egypt

## Abstract

Tuberculosis (TB) is the world's most prevalently infectious disease. Molecular mechanisms behind tuberculosis remain unknown. microRNA (miRNA) is involved in a wide variety of diseases. To validate the significant genes and miRNAs in the current sample, two messenger RNA (mRNA) expression profile datasets and three miRNA expression profile datasets were downloaded from the Gene Expression Omnibus (GEO) database. The differentially expressed (DE) genes (DEGs) and miRNAs (DE miRNAs) between healthy and TB patients were filtered out. Enrichment analysis was executed, and a protein-protein interaction (PPI) network was developed to understand the enrich pathways and hub genes of TB. Additionally, the target genes of miRNA were predicted and overlapping target genes were identified. We studied a total of 181 DEGs (135 downregulated and 46 upregulated genes) and two DE miRNAs (2 downregulated miRNAs) from two gene profile datasets and three miRNA profile datasets, respectively. 10 hub genes were defined based on high degree of connectivity. A PPI network's top module was constructed. The 23 DEGs identified have a significant relationship with miRNAs. 25 critically significant Gene ontology (GO) and Kyoto Encyclopedia of Genes and Genomes (KEGG) pathways were discovered. The detailed study revealed that, in tuberculosis, the DE miRNA and DEGs form an interaction network. The identification of novel target genes and main pathways would aid with our understanding of miRNA's function in tuberculosis progression.

## 1. Introduction

Tuberculosis (TB) is one of the most common infectious diseases, caused by the pathogen *Mycobacterium tuberculosis* (MTB). It was linked to a high rate of infection and a long-term disease course [1]. TB is one of the top 10 causes of death according to the World Health Organization (WHO)'s global TB report for 2020. Each year, about ten million people were infected with tuberculosis. TB was identified as a repetitive immune reaction, and a subset of patients with tuberculosis will grow into active tuberculosis. It has a close connection with poverty. Around 90% of tuberculosis patients stayed in underdeveloped countries [2]. There are many diagnostic tools available today, including the tuberculin skin examination (TST), the interferon gamma release assay (IGRA), and imaging procedures. They all lack precision and are more expensive and highly technical [3, 4].

microRNAs (miRNAs) are noncoding RNA molecules that have 22 to 23 nucleotides [5]. They regulate gene expression at the posttranscriptional stage by facilitating mRNA degradation and preventing mRNA translation [6]. When a patient is infected with tuberculosis, major miRNAs are released into the bloodstream. MiRNAs have shown to play an important role in a variety of pathological and physiological mechanisms in tuberculosis [7]. Microarray analysis, miRNA, and gene have been extensively utilized in the quest for biomarkers and therapeutic targets in recent years. The discovery of novel DE miRNAs and DEGs provides useful and accurate perspectives for future study [8].

To validate the significant genes and miRNAs, two mRNA expression profile datasets, GSE62147 [9] and GSE34608-GPL6480 [10], and three miRNA expression profile datasets, GSE34608-GPL7731 [10], GSE29190 [11], and GSE49951 [12], were used. The 250 DEGs in speech profile datasets were, respectively, identified. The enrichment analysis of DEGs showed 5 KEGG pathways were related to the development and progress of TB, including ‘hepatocellular carcinoma,' ‘Kaposi sarcoma-associated herpesvirus infection,' ‘phosphatidylinositol signaling system,' ‘circadian entrainment,' and ‘apelin signaling pathway.' A protein-protein interaction (PPI) network of DEGs and DEGs-DE miRNAs was created.10 hub genes were then chosen.

The present study established two DE miRNAs, hsa-mir-7 and hsa-mir-451. The gene-encoding hsa-mir-7 and hsa-mir-451 were separately located in human chromosomal region 9q21.32 and 17qll.2. The recent documents presented that Hsa-mir-7 and hsa-mir-451were involved in cancer-related biological processes and innate immune response [13, 14]. But, their future roles in TB remain unknown.

## 2. Materials and Methods

### 2.1. Microarray Data Screening

**Figure 1:**
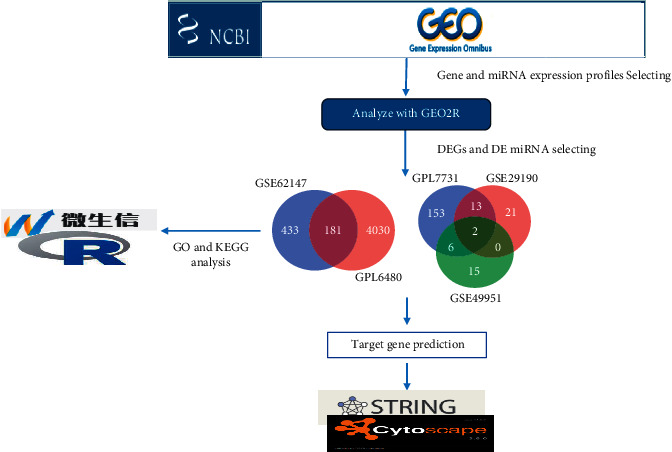
Flow diagram of bioinformatics analysis.

The bioinformatics analysis was carried out in accordance with the protocol depicted in Figure 1. The term 'Tuberculosis” or “microRNA and Tuberculosis” was searched in the Gene Expression Omnibus database (GEO). There are some database criteria that are helpful with more analysis: I) clinical study pairs must have both healthy and tuberculosis patients, and II) mRNA and miRNA data from plasma (Affymetrix miRNA 4.0) were retrieved. As a result, two gene expression profile datasets, GSE62147 and GSE34608-GPL6480, were used in the primal mRNA datasets. GSE62147 had 14 TB-positive donors and 14 healthy donors. GSE34608-GPL6480 had eight TB donors and eighteen healthy donors. Six donors with tuberculosis and three healthy donors contained GSE29190. GSE49951 had 71 TB-positive donors and 71 healthy donors.

### 2.2. Selecting DEGs and DE miRNAs and Constructing a Volcanic Map

To diagnose DEGs and DE miRNAs in TB patients, the GEO2R online research method (https://www.ncbi.nlm.nih.gov/geo/geo2r/) was used. Exact cutoff values (*P* < 0.05 and |log fold change (FC)|≥1) were established. The two gene and three miRNA datasets were submitted to VENN's online tool (http://bioinformatics.psb.ugent.be/webtools/Venn/). The DEGs and DE miRNAs that overlap were filtered out. The volcanic chart of overlapping DEGs was constructed using the online method of bioinformatics (http://www.bioinformatics.com.cn/).

### 2.3. GO and KEGG Pathway analysis

Gene Ontology (Go) was applied to perform the category biological process (BP), molecular function (MF), and cellular component (CC) enrichment analysis [15]. The KEGG was a set of databases that provides information regarding biological mechanisms, cellular processes, chemical substances, and diseases [16]. The R software package clusterProfiler was used in functional gene annotations and KEGG enrichment analysis [17]. A cutoff criterion (*P* < 0.05 and FDR<0.05) was set. Also, the top 10 pathways with the maximum number of genes for the corresponding term were chosen. Significant items of GO and KEGG were submitted to R software package ggplot2 to visualize and merge enriching analysis. The enrichment dot bubble method was used to build the bubble plot.

### 2.4. Construction of Network and Identification of Top Modules and Hub Genes

Permanent DEGs were uploaded to the STRING online tool (https://string-db.org/) [18]. A cutoff point (interaction score >0.4) was established. The PPI network was constructed using the CytoScape program [19]. The MCODE plug-in was used to determine the PPI network's top module. Strict cutoff conditions (degree cutoff = 2, node score cutoff = 0.2, *k*-core = 2, and maxdepth = 100) were established. Meanwhile, the top 10 hub genes were identified using the CytoHubba plug-in.

### 2.5. Identification of the Target Gene and Construction of the DE miRNA-DEGs Regulatory Network

To predict miRNA target genes, the online tools TargetScan (http://www.targetscan.org/vert71) [20], miRDB (http://www.mirdb.org/) [21], miRWalk (http://mirwalk.uni-hd.de/) [22], and miRTarBase (http://mirtarbase.cuhk.edu.cn/) [21] were used. The criterion for goal gene selection is cumulative weighted context++ score online > -0.5. The target gene that met the criteria was chosen from three databases. The overlapping target gene of miRNA was discovered using the VENN online method. CytoScape was used to upload miRNA and DEGs. To visualize and merge analysis, a DE miRNA-DEGs regulatory network was developed.

## 3. Results

### 3.1. Screening of DEGs and DE miRNAs

**Figure 2:**
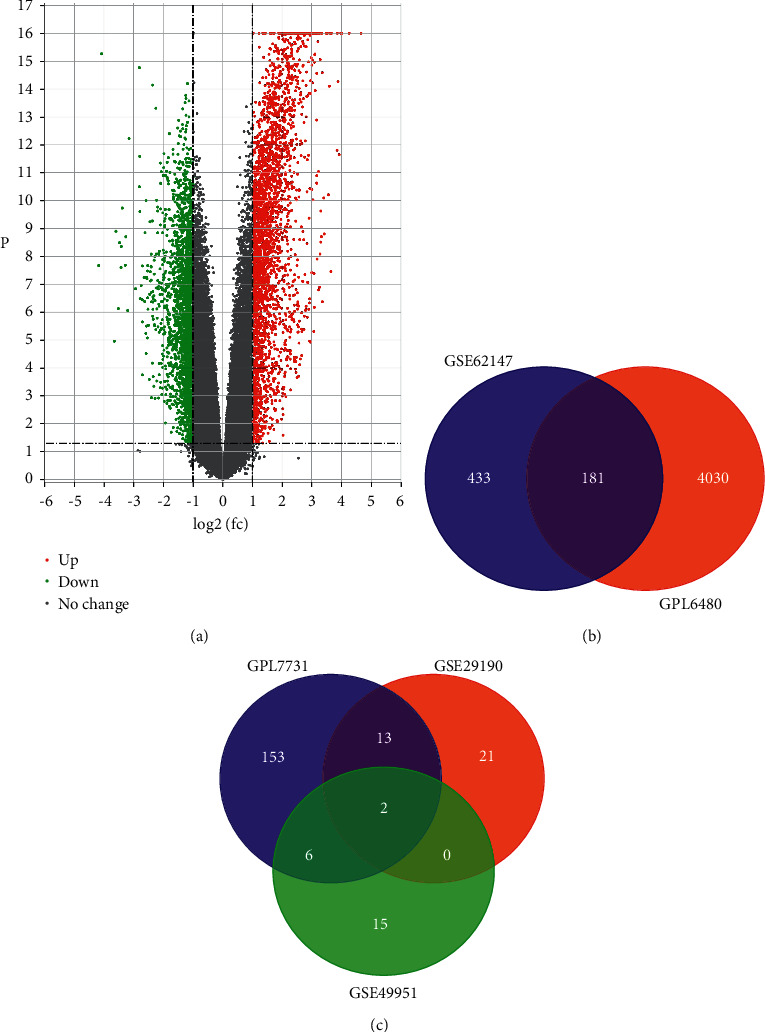
(a) Volcano plot showing downregulated and upregulated differentially expressed genes. (b) Venn diagram displaying the number of common differentially expressed genes (DEGs) between normal people and patients with TB. (c) Venn diagram for common differentially expressed miRNA (DE miRNA).

Under the precise cutoff criteria employed (*P* < 0.05 and |log fold change (FC)|≥1), there were 614 and 4211 DEGs extracted from GSE6214 and GSE34608-GPL6480, respectively. Following that, DE miRNAs were extracted from GSE34608-GPL7731, GSE29190, and GSE49951, respectively, yielding 174, 36, and 23 DE miRNAs. Two gene expression profile datasets yielded a total of 181 overlapping DGEs (135 downregulated genes and 46 upregulated genes) (Figure 2(b) and Table 1). Two overlapping DE miRNAs (two downregulated and zero upregulated) were derived from three miRNA expression profile datasets (Figure 2(c) and Table 2). The volcano plot of three gene expression profile datasets revealed a substantial difference between regular and tuberculosis patients (Figure 2(a)).

### 3.2. GO Functional and KEGG Pathway Enrichment analysis

**Figure 3:**
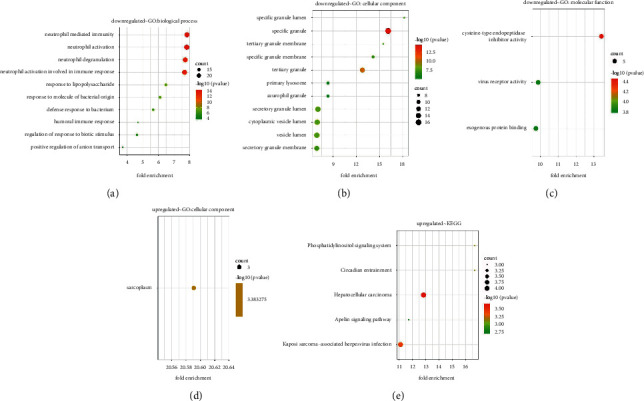
The gene ontology (GO) and Kyoto Encyclopedia of Genes and Genomes (KEGG) pathway enrichment analysis of differentially expressed genes. The biological process (BP), bellular component (CC), and molecular function (MF) consist of GO enrichment analysis commonly. (a) The enrichment dot bubble of GO-BP analysis, (b) the enrichment dot bubble of GO-CC analysis, and (c) the enrichment dot bubble of GO-MF analysis derived from downregulated DEGs. (d) The enrichment dot bubble of GO analysis derived from upregulated DEGs. (e) The enrichment dot bubble of KEGG analysis derived from DEGs.

DEGs that were consistent were submitted to R software for GO functional and KEGG pathway enrichment analysis. GO-CC showed that upregulated DEGs were mainly associated with ‘sarcoplasm' and downregulated DEGs were mainly associated with ‘specific granule,' ‘tertiary granule,' ‘secretory granule lumen,' ‘cytoplasmic vesicle lumen,' ‘vesicle lumen,' ‘secretory granule membrane,' ‘specific granule membrane,' ‘specific granule lumen,' ‘tertiary granule membrane,' ‘primary lysosome,' and ‘azurophil granule.' GO-BP showed that downregulated DEGs were primarily involved in ‘neutrophil-mediated immunity,' ‘neutrophil activation,' ‘neutrophil degranulation,' ‘neutrophil activation involved in immune response,' ‘response to lipopolysaccharide,' ‘response to a molecule of bacterial origin,' ‘defense response to a bacterium,' ‘regulation of response to biotic stimulus,' ‘humoral immune response,' and ‘positive regulation of anion transport.' GO-MF showed that downregulated DEGs were mainly related with ‘cysteine-type endopeptidase inhibitor activity,' ‘virus receptor activity,' and ‘exogenous protein binding' (Figure 3(a)–3(d) and Table 3).

The result represented that upregulated DEGs were significantly associated with 5 KEGG pathways, including ‘hepatocellular carcinoma,' ‘Kaposi sarcoma-associated herpesvirus infection,' ‘phosphatidylinositol signaling system,' ‘circadian entrainment,' and ‘apelin signaling pathway' (Figure 3(e), Table 4).

### 3.3. Hub Gene Identification Based on the DEG PPI Network and Module Analysis

**Figure 4:**
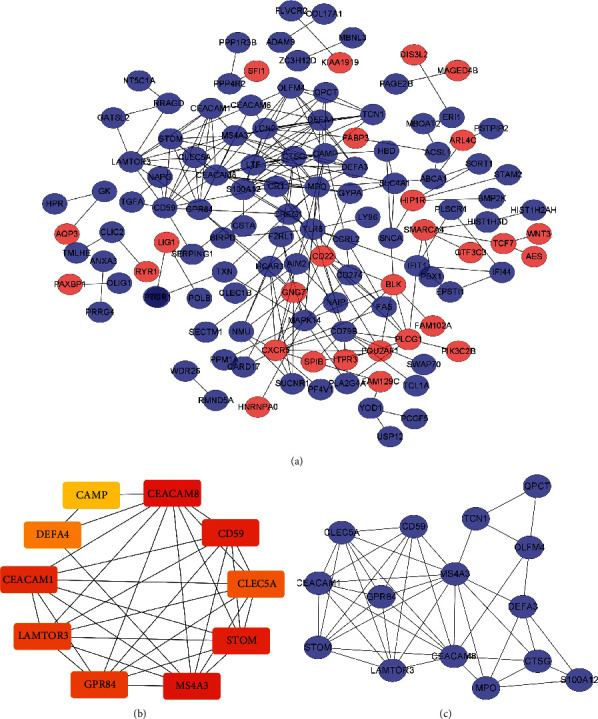
(a) Circular nodes show the DEGs. The blue nodes stand for downregulated DEGs, and red nodes stand for upregulated DEGs. (b) The 10 hub genes identified from the PPI network. (c) The top module extracted from the PPI network. The blue nodes stand for downregulated DEGs, and red nodes stand for upregulated DEGs.

The STRING online tool was updated with 181 common DEGs (135 downregulated and 46 upregulated). Significant DEGs were identified in 135 of 181 DEGs. Significant DEGs were visualized in detail using the CytoScape software. The PPI network was constructed with 122 nodes and 241 edges (Figure 4(a)). Additionally, 59 of 181 DEGs were not part of the PPI network. The hub genes contained Cathelicidin Antimicrobial Peptide (CAMP), CEA Cell Adhesion Molecule 8 (CEACAM8), CD59 molecule (CD59), C-Type Lectin Domain Containing 5A (CLEC5A), Stomatin (STOM), Membrane-Spanning 4-Domains A3 (MS4A3), G Protein-Coupled Receptor 84 (GPR84), Late Endosomal/Lysosomal Adaptor, MAPK and MTOR Activator 3 (LAMTOR3), Defensin Alpha 4 (DEFA4), and CEA Cell Adhesion Molecule 1 (CEACAM1) (Figure 4(b)). The top significant module was defined from the PPI network using the MCODE plug-in based on its degree value. The top module featured fifteen nodes and forty-five corners (Figure 4(c)).

### 3.4. Construction of the DEGs-DE miRNA Network

**Figure 5:**
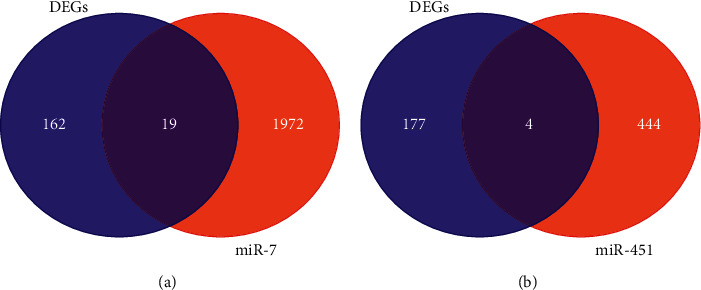
(a) VENN diagram identified the consistent genes between the DEGs and the target genes of miRNA-7. (b) VENN diagram identified the consistent genes between the DEGs and the target genes of miRNA-451.

**Figure 6:**
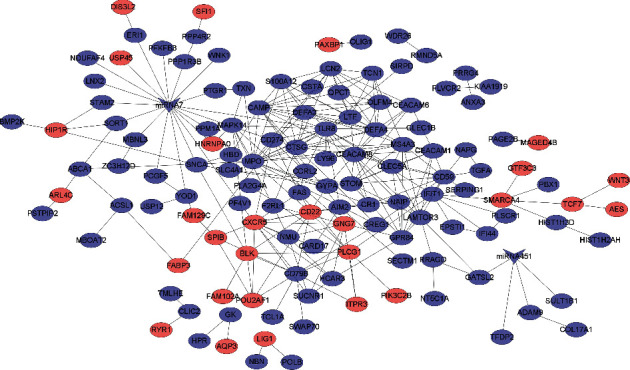
The PPI network of DEGs and DE miRNA. The blue nodes indicate the downregulated genes. The red nodes indicated the upregulated genes. The blue V-shape nodes stand for downregulated miRNA.

Because of the shortcomings of each dataset, hsa-mir-7 and hsa-mir-451 were each submitted to four accurate datasets for target gene prediction. Using the VENN online research website, miRNA target genes were integrated with DEGs. The identified data revealed that hsa-target mir-7 genes shared 19 common genes with DEGs (Figure 5(a)) and the hsa-goal mir-451 gene shared four genes with DEGs (Figure 5(b)). Meanwhile, CytoScape developed the DEGs-DE miRNA PPI network (Figure 6).

## 4. Discussion

In recent years, a number of studies have been carried out to reveal the potential mechanisms of tuberculosis. The prevalence of tuberculosis has been steadily rising. Traditional studies have two flaws: a single genetic case and a limited cohort [23]. Bioinformatics analysis of gene expression profile datasets of TB patients is now being used to screen more reliable data. A total of 181 common DEGs (135 downregulated genes and 46 upregulated genes) were extracted from two gene expression profile datasets in this study. The hsa-mir-7 and hsa-mir-451, two DE miRNAs, were extracted from four gene expression profile datasets. Both hsa-mir-7 and hsa-mir-451 were reduced in expression. The 181 DEGs underwent GO and KEGG enrichment analysis, allowing them to be classified into BP, CC, MF, and KEGG groups. Finally, a DEGs-DE miRNA PPI network was built. The PPI network was used to screen the top module and ten hub genes.

To determinate the underlying molecular mechanisms in the TB process, the most enriched BP, CC, and MF pathways were combined with downregulated and upregulated DEGs for comprehensive analysis, separately. GO analysis presented that upregulated DEGs were mainly related with sarcoplasm and downregulated DEGs were mainly related with neutrophil, antibacterial activities, primary lysosome, and exogenous protein binding. The comprehensive analysis of GO and hub genes demonstrated that hub genes mainly associated with neutrophil and primary lysosome signal pathways. KEGG analysis presented that upregulated DEGs were mostly involved in hepatocellular carcinoma, Kaposi sarcoma-associated herpesvirus infection, the phosphatidylinositol signaling system, circadian entrainment, and the apelin signaling pathway. Most of the abovementioned KEGG pathways play an important role in the immune response and apoptosis of *Mycobacterium tuberculosis*. Kaposi sarcoma-associated herpesvirus is well known to be involved in antiapoptosis, enhancement of cytokine production, and cell proliferation [24]. The previous study reported that MTB enhances bacterial virulence by avoiding host cell death [25]. Phosphatidylinositol is a lipid anchor, which as virulence factor and modulate host immune response in MTB [26]. Also, the apelin decreases mitochondrial apoptosis, mitochondrial ROS-triggered oxidative damage, and NF-*κ*B activation to inhibit acute lung injury (ALI) and acute respiratory distress syndrome (ARDS) [27]. Circadian entrainment was also involved in innate immune response and photoperiod significant impairment and enhancing immune function [28].

According to a recent study, hsa-mir-7 impaired NF-*κ*B and AKT transcriptional activity. The NF-*κ*B and AKT pathways are important inflammatory-associated pathways and inhibited host cell autophagy in tuberculosis [4, 29, 30]. The hsa-mir-7 was thought to be involved in innate immune responses in a previous study [31]. Meanwhile, hsa-mir-451 protects against cell death caused by ischemia/reperfusion injury, cancer, and myocardial I/R injury [32–34]. CAMP, CEACAM8, CD59, CLEC5A, STOM, MS4A3, GPR84, LAMTOR3, CEACAM1, and DEFA4 are among the ten hub genes discovered. They act as TB immune mediators and, additionally, as an active participant in the control of the inflammatory response [35–40]. Meanwhile, macrophages are innate immune cells that serve as a guardian in the immune system's protection and response to microbial infection [41]. GPR84 is a member of the family of metabolic G protein-coupled receptors [42]. GPR84 activation enhances bacterial adhesion and phagocytosis in macrophages. According to a recent study, elevated glucose levels promote GPR84 expression [43]. STOM is a member of the family of integral membrane proteins [44]. As previously mentioned, STOM's primary role is to regulate glucose transporter type 1 operation. Furthermore, elevated glucose levels augment macrophage anti-inflammatory function [45]. GPR84 and STOM are closely associated with the adaptive immune response, as determined by experimentation. CAMP is an antimicrobial peptide that is synthesized at the C-terminus of proteins [46]. It eradicated MTB and slowed the progression of tuberculosis. CAMP is a vitamin D-related gene that would be triggered in response to vitamin D. It is essential for macrophages infected with MTB to benefit from the antimicrobial reaction induced by vitamin D. CAMP activation enhances the effectiveness of immune responses in tuberculosis [47]. CEACAM1 and CEACAM8 are members of the class of heavily glycosylated carcinoembryonic antigens [48, 49]. The primary role of CEACM1 is antiapoptotic. It is involved in granulocyte survival [50]. It is classified as an immunoregulatory checkpoint regulator. Previous research demonstrated that inadequate CEACAM1 resulted in inflammatory exacerbation [51]. CEACM8 is a responsive granulocyte biomarker. It performs two functions: it recognizes neutrophils and degrades extracellular matrix, thus stimulating the immune response [52]. MTB has been shown to control the immune system by controlling the macrophage cell cycle. It prevents macrophages from entering the interphase and gap phase 1 phases [53]. MS4A3 is a member of the family of membrane-spanning 4A genes [54]. MS4A3 is intimately linked to the cell cycle. As a consequence, the activation of the kinase-associated phosphatase (KAP) leads to cell cycle arrest. CLEC5A is a member of the superfamily C-type lectin/C-type lectin-like domain (CTL/CTLD) [54]. CLEC5A controls cell development by inducing apoptosis and arresting the cell cycle. Additionally, it plays a critical function in the production of inflammation and serves as a critical gene for the clinical management of pulmonary inflammation [55]. DEFA4 is a part of the lipocalin family, which is involved in the transportation of vitamins, lipids, and steroid hormones [56]. It inhibits bacterial growth by attaching to pathogenic bacterial siderophores. It plays a significant role in immune response and protection. In recent years, LAMTOR3 and CD59 have been extensively used to inhibit the development of a variety of cancers [57, 58]. CD59's primary role is to regulate immune cell activation throughout the tumor microenvironment [59]. Additionally, the top module was defined through the PPI network of DEGs. The top module included a large number of genes associated with macrophages and the innate immune response. Previously published studies established that macrophages act as a host cell for MTB and are active in the innate immune response. MTB destroyed the macrophage's main immune systems, antigen introduction and intracellular killing [41].

The important DEGs were established using integrated bioinformation analysis. GPR84, STOM, CAMP, CEACM8A, MS4A3, LAMTOR3, DEFA4, CLEC5A, and CD59 were the ten hub genes that were filtered out. Two DE miRNAs, hsa-mir-7 and hsa-mir-451, were discovered. Some important pathways, including neutrophil, antibacterial activities, primary lysosome, exogenous protein binding, the apelin signaling pathway, Kaposi sarcoma-associated herpesvirus infection, and the phosphatidylinositol signaling system, are involved in TB. DEGs and DE miRNAs may be used as sensitive biomarkers in the clinical diagnosis of tuberculosis. The network of DE miRNA-DEGs revealed the molecular mechanism causing tuberculosis. More accurate clinical samples and experiments are needed to validate the cause of tuberculosis. In the future, bioinformatics research could be able to identify novel genes and pathways for use in genomic therapy for tuberculosis.

## Figures and Tables

**Table 1:** Differentially expressed genes between the normal people and patients with TB.

Regulations	DEGs
Upregulated	LHFPL2, ANXA3, F2RL1, PPM1A, VWCE, LAMTOR3, SECTM1, GYPA, SNCA, TDRD9, WDR26, ALPK1, CR1, RMND5A, FAM8A1, OLFM4, ACSL1, GPR84, MBNL3, ARG2, PPP4R2, HIST1H3D, CLEC5A, STAM2, ABCC13, S100A12, C2orf88, TMLHE, BMP2K, SAMD4B, LY96, TCN1, AEBP1, HIST1H2AH, DDX60L, HEMK1, LCN2, DOK7, CLCF1, MBOAT2, NDUFAF4, CEACAM6, SERPING1, PBX1, GATSL2, OLIG1, CD79B, TCL1A, PLSCR1, PF4V1, ZNF438, NMU, SUCNR1, DEFA4, GUSBP3, SWAP70, HCAR3, HBD, CDHR3, MIAT, LGALSL, PCGF5, ERV3-2, ZC3H12D, ZNF451, CREG1, IFIT1, LTF, TGFA, LNX2, ABLIM3, CLIC2, SULT1B1, IFI44, PIK3IP1, NT5C1A, CD274, TXN, EFCAB2, PTGR1, DEFA3, NSUN3, PFKFB3, PPP1R3B, ABCA1, GK, ERI1, TSTA3, NBN, IL18R1, CEACAM1, CARD17, USP12, LINC01232, MS4A3, MCTP2, EPSTI1, NAIP, ANKRD22, CSTA, LOXL1, SORT1, CLEC1B, SLC4A1, ZAK, WNK1, AIM2, RRAGD, COL17A1, BEX1, FLVCR2, TFDP2, YOD1, NAPG, CEACAM8, FAS, PRRG4, ABCC4, PAGE2B, MAPK14, HPR, STOM, PLA2G4A, MPO, TLR8, ADAM9, CAMP, QPCT, SIRPD, POLB, PSTPIP2, CCRL2, CD59, CTSG, and AP5B1
Downregulated	ZNF683, FAM193B, DIS3L2, SPIB, GUSBP4, EVL, CD22, PAXBP1, LIG1, SFI1, AQP3, FAM102A, TNFRSF25, HNRNPA0, GUSBP1, FCGBP, WNT3, RYR1, GTF3C3, AES, ITPR3, CRIP3, TCF7, MFSD4B, LOC105370792, MIR600HG, FAM129C, BLK, SMG1P5, PIK3C2B, SMARCA4, GNG7, HIP1R, PLCG1, LOC100132363, POU2AF1, STMN3, ARL4C, FABP3, USP45, SUN2, DMRTC1, CXCR5, EPPK1, MAGED4B, and SNX29P2

DEGs, differentially expressed genes; TB, tuberculosis

**Table 2:** Differentially expressed miRNA between the normal people and patients with TB.

Regulation	*n*	name
Upregulated	0	
Downregulated	2	miRNA-7 miRNA-451

**Table 3:** Gene ontology analysis of common upregulated and downregulated DEGs.

Category	Term	Count	Gene ratio	*P* value
*Upregulated*				
CC	GO:0016528∼sarcoplasm	3	8.33*E* − 02	4.14*E* − 04
*Downregulated*				
BP	GO:0002446∼neutrophil-mediated immunity	24	2.07*E* − 01	3.68*E* − 15
BP	GO:0042119∼neutrophil activation	24	2.07*E* − 01	3.85*E* − 15
BP	GO:0043312∼neutrophil degranulation	23	1.98*E* − 01	2.01*E* − 14
BP	GO:0002283∼neutrophil activation involved in immune response	23	1.98E-01	2.29*E* − 14
BP	GO:0032496∼response to lipopolysaccharide	13	1.12*E* − 01	1.08*E* − 07
BP	GO:0002237∼response to a molecule of bacterial origin	13	1.12*E* − 01	2.14*E* − 07
BP	GO:0042742∼defense response to a bacterium	12	1.03*E* − 01	1.41*E* − 06
BP	GO:0002831∼regulation of response to a biotic stimulus	12	1.03*E* − 01	1.08*E* − 05
BP	GO:0006959∼humoral immune response	11	9.48*E* − 02	2.28*E* − 05
BP	GO:1903793∼positive regulation of anion transport	11	9.48*E* − 02	1.77*E* − 04
MF	GO:0004869∼cysteine-type endopeptidase inhibitor activity	5	4.10*E* − 02	3.50*E* − 05
MF	GO:0001618∼virus receptor activity	5	4.10*E* − 02	1.52*E* − 04
MF	GO:0140272∼exogenous protein binding	5	4.10*E* − 02	1.62*E* − 04
CC	GO:0042581∼specific granule	16	1.32*E* − 01	3.31*E* − 15
CC	GO:0070820∼tertiary granule	13	1.07*E* − 01	2.88*E* − 11
CC	GO:0034774∼secretory granule lumen	14	1.16*E* − 01	1.29*E* − 08
CC	GO:0060205∼cytoplasmic vesicle lumen	14	1.16*E* − 01	1.51*E* − 08
CC	GO:0031983∼vesicle lumen	14	1.16*E* − 01	1.63*E* − 08
CC	GO:0030667∼secretory granule membrane	13	1.07*E* − 01	5.56*E* − 08
CC	GO:0035579∼specific granule membrane	8	6.61*E* − 02	9.55*E* − 08
CC	GO:0035580∼specific granule lumen	7	5.79*E* − 02	1.09*E* − 07
CC	GO:0070821∼tertiary granule membrane	7	5.79*E* − 02	3.43*E* − 07
CC	GO:0005766∼primary lysosome	8	6.61*E* − 02	5.56*E* − 06
CC	GO:0042582∼azurophil granule	8	6.61*E* − 02	5.56*E* − 06

GO, Gene Ontology; DEGs, differentially expressed genes; BP, biological process; CC, cellular component; MF, molecular function; Count, number of DEGs.

**Table 4:** KEGG pathway analysis of common upregulated DEGs.

Pathway	Id	Count	Fold enrichment	*P* value	Genes
*Upregulated*					
Hepatocellular carcinoma	hsa05225	4	12.846	2.05*E* − 04	WNT3, TCF7, SMARCA4, and PLCG1
Kaposi sarcoma-associated herpesvirus infection	hsa05167	4	11.124	3.55*E* − 04	ITPR3,TCF7,GNG7, and PLCG1
Phosphatidylinositol signalling system	hsa04070	3	16.687	6.84*E* − 04	ITPR3, PIK3C2B, and PLCG1
Circadian entrainment	hsa04713	3	16.687	6.84*E* − 04	RYR1, ITPR3, and GNG7
Apelin signaling pathway	hsa04371	3	11.729	1.90*E* − 03	RYR1, ITPR3, and GNG7

KEGG, Kyoto Encyclopedia of Genes and Genomes; DEGs, differentially expressed genes; Count, number of DEGs.

## Data Availability

There are no files that need to be deposited in public repositories among the data in the manuscript. The manuscript contains all necessary information about the data collected.

## References

[1] Dheda K. (2016). Tuberculosis and Poncet’s disease: the many faces of an old enemy. *The Lancet*.

[2] Strausz J. (2007). Tuberculosis. *Orvosi Hetilap*.

[3] Cavusoglu C. (2017). [Evaluation of QuantiFERON(R)-TB gold in tube test and tuberculin skin test in the diagnosis of *Mycobacterium tuberculosis* infection]. *Mikrobiyoloji Bulteni*.

[4] Zhou X., Li J., Zhou Y. (2020). Down-regulated ciRS-7/up-regulated miR-7 axis aggravated cartilage degradation and autophagy defection by PI3K/AKT/mTOR activation mediated by IL-17A in osteoarthritis. *Aging*.

[5] Huang W. (2017). MicroRNAs: biomarkers, diagnostics, and therapeutics. *Bioinformatics in MicroRNA Research*.

[6] Correia D. S. M (2019). Deciphering miRNAs’ action through miRNA editing. *International Journal of Molecular Sciences*.

[7] Backes C., Meese E., Keller A. (2016). Specific miRNA disease biomarkers in blood, serum and plasma: challenges and prospects. *Molecular Diagnosis and Therapy*.

[8] Zheng Y., Qing T., Song Y. (2015). Standardization efforts enabling next-generation sequencing and microarray based biomarkers for precision medicine. *Biomarkers in Medicine*.

[9] Tientcheu L. D., Maertzdorf J., Weiner J. (2015). Differential transcriptomic and metabolic profiles of M. africanum- and M. tuberculosis-infected patients after, but not before, drug treatment. *Genes and Immunity*.

[10] Maertzdorf J., Weiner J., Mollenkopf H.-J. (2012). Common patterns and disease-related signatures in tuberculosis and sarcoidosis. *Proceedings of the National Academy of Sciences*.

[11] Wang C., Yang S., Sun G. (2011). Comparative miRNA expression profiles in individuals with latent and active tuberculosis. *PLoS One*.

[12] Siddle K. J., Deschamps M., Tailleux L. (2014). A genomic portrait of the genetic architecture and regulatory impact of microRNA expression in response to infection. *Genome Research*.

[13] Bai H., Wu S. (2019). miR-451: a novel biomarker and potential therapeutic target for cancer. *OncoTargets and Therapy*.

[14] Alamdari-Palangi V., Karami Z., Karami H., Baazm M. (2020). MiRNA-7 replacement effect on proliferation and tarceva-sensitivity in U373-MG cell line. *Asian Pacific Journal of Cancer Prevention*.

[15] Ashburner M., Ball C. A., Blake J. A. (2000). Gene Ontology: tool for the unification of biology. *Nature Genetics*.

[16] Kanehisa M., Goto S. (2000). KEGG: kyoto encyclopedia of genes and genomes. *Nucleic Acids Research*.

[17] Zhang M., Zhu K., Pu H. (2019). An immune-related signature predicts survival in patients with lung adenocarcinoma. *Frontiers in Oncology*.

[18] Szklarczyk D., Gable A. L., Lyon D. (2019). STRING v11: protein-protein association networks with increased coverage, supporting functional discovery in genome-wide experimental datasets. *Nucleic Acids Research*.

[19] Shannon P. (2003). Cytoscape: a software environment for integrated models of biomolecular interaction networks. *Genome Research*.

[20] Peterson S. M., Thompson J. A., Ufkin M. L., Sathyanarayana P., Liaw L., Congdon C. B. (2014). Common features of microRNA target prediction tools. *Frontiers in Genetics*.

[21] Weng W., Zhang Z., Huang W. (2020). Identification of a competing endogenous RNA network associated with prognosis of pancreatic adenocarcinoma. *Cancer Cell International*.

[22] Sticht C., De La Torre C., Parveen A., Gretz N. (2018). miRWalk: an online resource for prediction of microRNA binding sites. *PLoS One*.

[23] Huang D. W., Sherman B. T., Lempicki R. A. (2009). Bioinformatics enrichment tools: paths toward the comprehensive functional analysis of large gene lists. *Nucleic Acids Research*.

[24] Gonçalves P. H., Uldrick T. S., Yarchoan R. (2017). HIV-associated Kaposi sarcoma and related diseases. *AIDS*.

[25] Briken V., Miller J. L. (2008). Living on the edge: inhibition of host cell apoptosis by *Mycobacterium tuberculosis*. *Future Microbiology*.

[26] Belcher Dufrisne M., Jorge C. D., Timóteo C. G. (2020). Structural and functional characterization of phosphatidylinositol-phosphate biosynthesis in mycobacteria. *Journal of Molecular Biology*.

[27] Yan J., Wang A., Cao J., Chen L. (2020). Apelin/APJ system: an emerging therapeutic target for respiratory diseases. *Cellular and Molecular Life Sciences*.

[28] Onishi K. G., Maneval A. C., Cable E. C. (2020). Circadian and circannual timescales interact to generate seasonal changes in immune function. *Brain, Behavior, and Immunity*.

[29] He W., Sun J., Zhang Q. (2020). Andrographolide exerts anti‐inflammatory effects in *Mycobacterium tuberculosis* ‐infected macrophages by regulating the Notch1/Akt/NF‐*κ*B axis. *Journal of Leukocyte Biology*.

[30] Ye T., Yang M., Huang D. (2019). MicroRNA-7 as a potential therapeutic target for aberrant NF-*κ*B-driven distant metastasis of gastric cancer. *Journal of Experimental & Clinical Cancer Research*.

[31] Zhao J., Chen C., Guo M. (2016). MicroRNA-7 deficiency ameliorates the pathologies of acute lung injury through elevating KLF4. *Frontiers in Immunology*.

[32] Gilfillan M., Das P., Shah D., Alam M. A., Bhandari V. (2020). Inhibition of microRNA-451 is associated with increased expression of Macrophage Migration Inhibitory Factor and mitigation of the cardio-pulmonary phenotype in a murine model of Bronchopulmonary Dysplasia. *Respiratory Research*.

[33] Liu Q., Hu Y., Zhang M., Yan Y., Yu H., Ge L. (2018). microRNA-451 protects neurons against ischemia/reperfusion injury-induced cell death by targeting CELF2. *Neuropsychiatric Disease and Treatment*.

[34] Kong W., Feng L., Yang M. (2019). Prognostic value of microRNA-451 in various cancers: a meta-analysis. *Pathology, Research & Practice*.

[35] Zhang R., Liu Q., Liao Q., Zhao Y. (2018). CD59: a promising target for tumor immunotherapy. *Future Oncology*.

[36] Ribon M., Mussard J., Semerano L., Singer B. B., Decker P. (2019). Extracellular chromatin triggers release of soluble CEACAM8 upon activation of neutrophils. *Frontiers in Immunology*.

[37] Wojciechowicz M. L., Ma’ayan A. (2020). GPR84: an immune response dial?. *Nature Reviews Drug Discovery*.

[38] Sprokholt J., Helgers L. C., Geijtenbeek T. B. (2017). Innate immune receptors drive dengue virus immune activation and disease. *Future Virology*.

[39] Silva S., Santos-Silva A., da Costa J. M. C., Vale N. (2019). Potent cationic antimicrobial peptides against *Mycobacterium tuberculosis* in vitro. *Journal of Global Antimicrobial Resistance*.

[40] Mukherjee S., Sarkar-Roy N., Wagener D. K., Majumder P. P. (2009). Signatures of natural selection are not uniform across genes of innate immune system, but purifying selection is the dominant signature. *Proceedings of the National Academy of Sciences*.

[41] Hmama Z., Peña-Díaz S., Joseph S., Av-Gay Y. (2015). Immunoevasion and immunosuppression of the macrophage byMycobacterium tuberculosis. *Immunological Reviews*.

[42] Wittenberger T., Schaller H. C., Hellebrand S. (2001). An expressed sequence tag (EST) data mining strategy succeeding in the discovery of new G-protein coupled receptors11Edited by J. Thornton. *Journal of Molecular Biology*.

[43] Recio C., Lucy D., Purvis G. S. D. (2018). Activation of the immune-metabolic receptor GPR84 enhances inflammation and phagocytosis in macrophages. *Frontiers in Immunology*.

[44] Hiebl-Dirschmied C. M., Entler B., Glotzmann C., Maurer-Fogy I., Stratowa C., Prohaska R. (1991). Cloning and nucleotide sequence of cDNA encoding human erythrocyte band 7 integral membrane protein. *Biochimica et Biophysica Acta (BBA) - Gene Structure and Expression*.

[45] An H., au fnm, Ma X. (2019). Stomatin plays a suppressor role in non-small cell lung cancer metastasis. *Chinese Journal of Cancer Research*.

[46] Gudmundsson G. H., Magnusson K. P., Chowdhary B. P., Johansson M., Andersson L., Boman H. G. (1995). Structure of the gene for porcine peptide antibiotic PR-39, a cathelin gene family member: comparative mapping of the locus for the human peptide antibiotic FALL-39. *Proceedings of the National Academy of Sciences*.

[47] Li Y., Østerhus S., Johnsen I. B. (2018). Human metapneumovirus infection inhibits Cathelicidin antimicrobial peptide expression in human macrophages. *Frontiers in Immunology*.

[48] Berling B., Kolbinger F, Grunert F (1990). Cloning of a carcinoembryonic antigen gene family member expressed in leukocytes of chronic myeloid leukemia patients and bone marrow. *Cancer Research*.

[49] Kuroki M., Arakawa F., Matsuo Y., Oikawa S., Nakazato H., Matsuoka Y. (1991). Three novel molecular forms of biliary glycoprotein deduced from cDNA clones from a human leukocyte library. *Biochemical and Biophysical Research Communications*.

[50] Singer B. B., Klaile E., Scheffrahn I. (2005). CEACAM1 (CD66a) mediates delay of spontaneous and Fas ligand-induced apoptosis in granulocytes. *European Journal of Immunology*.

[51] Horst A. K., Najjar S. M, Wagener C, Tiegs G (2018). CEACAM1 in liver injury, metabolic and immune regulation. *International Journal of Molecular Sciences*.

[52] Huang X., Pan Y., Ma J. (2018). Prognostic significance of the infiltration of CD163+macrophages combined with CD66b+neutrophils in gastric cancer. *Cancer Medicine*.

[53] Tipgomut C., Wongprommoon A., Takeo E., Ittiudomrak T., Puthong S., Chanchao C. (2018). Melittin induced G1 cell cycle arrest and apoptosis in chago-K1 human bronchogenic carcinoma cells and inhibited the differentiation of THP-1 cells into tumour- associated macrophages. *Asian Pacific Journal of Cancer Prevention*.

[54] Adra C. N., Lelias J. M., Kobayashi H. (1994). Cloning of the cDNA for a hematopoietic cell-specific protein related to CD20 and the beta subunit of the high-affinity IgE receptor: evidence for a family of proteins with four membrane-spanning regions. *Proceedings of the National Academy of Sciences*.

[55] Teng O., Chen S. T, Hsu T. L (2017). CLEC5A-Mediated enhancement of the inflammatory response in myeloid cells contributes to influenza virus pathogenicity in vivo. *Journal of Virology*.

[56] Palfree R. G., Sadro L. C., Solomon S. (1993). The gene encoding the human corticostatin HP-4 precursor contains a recent 86-base duplication and is located on chromosome 8. *Molecular Endocrinology*.

[57] Weinstock C., Anliker M., von Zabern I. (2015). CD59: a long-known complement inhibitor has advanced to a blood group system. *Immunohematology*.

[58] Lunin V. V., Munger C., Wagner J., Ye Z., Cygler M., Sacher M. (2004). The structure of the MAPK scaffold, MP1, bound to its partner, p14. *Journal of Biological Chemistry*.

[59] Chen J., Ding P., Li L. (2017). CD59 regulation by SOX2 is required for epithelial cancer stem cells to evade complement surveillance. *Stem Cell Reports*.

